# Correlation of Genomic and Pedigree Inbreeding Coefficients in Small Cattle Populations

**DOI:** 10.3390/ani11113234

**Published:** 2021-11-12

**Authors:** José Cortes-Hernández, Adriana García-Ruiz, Carlos Gustavo Vásquez-Peláez, Felipe de Jesus Ruiz-Lopez

**Affiliations:** 1 Centro Nacional de Investigación Disciplinaria en Fisiología y Mejoramiento Animal, Instituto Nacional de Investigaciones Forestales Agrícolas y Pecuarias, Km 1 Carretera a Colón, Ajuchitlán Colón, C.P., Querétaro 76280, Mexico; jgch1992@hotmail.com (J.C.-H.); garcia.adriana@inifap.gob.mx (A.G.-R.); 2 Departamento de Genética y Bioestadística, FMVZ-UNAM, Ciudad Universitaria, Av. Universidad #3000, Colonia C.U., Ciudad de México 04510, Mexico; carlosgv@unam.mx

**Keywords:** Holstein cattle, inbreeding coefficient, runs of homozygosity

## Abstract

**Simple Summary:**

This study aimed to evaluate the consistency of different methodologies and sources of information used to estimate inbreeding coefficients in small populations by analyzing the correlation between them in the Holstein population of Mexico and to choose the best option in order to aid breeding programs to improve the productive traits of Holstein cattle in small-specialized populations.

**Abstract:**

This study aimed to identify inbreeding coefficient (F) estimators useful for improvement programs in a small Holstein population through the evaluation of different methodologies in the Mexican Holstein population. F was estimated as follows: (a) from pedigree information (Fped); (b) through runs of homozygosity (Froh); (c) from the number of observed and expected homozygotic SNP in the individuals (Fgeno); (d) through the genomic relationship matrix (Fmg). The study included information from 4277 animals with pedigree records and 100,806 SNP. The average and standard deviation values of F were 3.11 ± 2.30 for Fped, −0.02 ± 3.55 for Fgeno, 2.77 ± 0.71 for Froh and 3.03 ± 3.05 for Fmg. The correlations between coefficients varied from 0.30 between Fped and Froh, to 0.96 between Fgeno and Fmg. Differences in the level of inbreeding among the parent’s country of origin were found regardless of the method used. The correlations among genomic inbreeding coefficients were high; however, they were low with Fped, so further research on this topic is required.

## 1. Introduction

For many years, the Holstein breed has dominated the dairy industry. Artificial insemination, which has been intensively used in dairy cattle since the 1980s [1], along with embryo transfer, are useful tools in genetic improvement that have increased the rate of genetic progress, decreased generation intervals, increased selection accuracy and reduced the costs of progeny testing [2]. All this, plus the use of genomic information has resulted in the selection of the next generation of animals from a reduced number of families or individuals with high genetic values [3], increasing inbreeding and, therefore, decreasing the effective population size. Furthermore, selection increases the frequency of homozygous regions in the genome, while at the same time decreasing the genetic diversity and affecting the phenotypic values for some productive features (inbreeding depression), lowering the yield per animal [4].

In small livestock populations, one of the most significant problems is the increase in inbreeding, the estimation of which is difficult and sometimes unreliable because of the lack of pedigree records [5].

The inbreeding coefficient (F) is defined as the probability that at any locus of an individual, genes identical by descent are found [6]; in other words, it is the replica of a gene at the moment the embryo is generated from two parents with one or more common ancestors [6].

Inbreeding coefficients can be estimated from different sources of information. Wright, in 1922 [6], stipulated a methodology based on pedigree information (Fped), where one obtains the probability of finding, at any locus of an individual, two identical copies of alleles coming from the same ancestral gene via both parental pathways; these are known as identical by descent genes. The estimation of that cumulative probability or inbreeding coefficient is calculated over all the common ancestors in the genealogy of an individual [7].

Recently, other methodologies based on the use of genetic markers in the DNA have been developed to estimate inbreeding coefficients; for example, one calculated with regions or runs of homozygosity (Froh; [8]), the genomic inbreeding coefficient (Fgeno; [9]), or the inbreeding estimated from the genomic relationship matrix (Fmg; [10]).

Runs of homozygosity (ROH) are contiguous DNA lengths of homozygotic genotypes present in an individual’s genome due to ancestors transmitting identical haplotypes from one generation to another. These regions can provide information about the level of inbreeding of an individual, its ancestry and the population it belongs to [11].

The ROH represent genomic homozygosity as a consequence of mating genomically related individuals; additionally, they may be able to reveal the regions that affect the fitness of an individual. Furthermore, these regions caused by inbreeding as an outcome of selection, increase the probability of undesirable recessive alleles being expressed [12]. Nevertheless, ROH can also be present due to unusual mutations, linkage disequilibrium, or a low recombination rate at certain genomic regions, sometimes resulting in a higher susceptibility to recessive illness [11].

The Fgeno is an inbreeding coefficient equivalent to Wright’s within-subpopulation fixation index. Values can range from −1 to +1. Negative numbers refer to outbreeding present in mating between individuals of different populations or less heterozygous than the population average. Positive values indicate the level of inbreeding of individuals of the same population [13].

The Fmg combines pedigree information with that from molecular makers. When only pedigree information is available, the offspring is assumed to randomly receive one-half of each parent’s genes. With the addition of genomic data, one can determine the proportion of the genes received from its ancestors, or in the case of individuals that share no known common ancestors, the proportion of common genes can be determined [14].

Inbreeding has effects on animal health and productivity, hence the possibility of using it as a tool to improve such characteristics. Nowadays, different options are available to estimate F, hence the importance of knowing if they are providing the same information.

One would expect that genomic information would provide a more accurate determination of the level of inbreeding in any individual compared to determination by pedigree. However, not all inbreeding is equal or expected to have adverse effects on fitness. More recent inbreeding is sometimes more detrimental to the productive and reproductive traits of cattle than ancestral inbreeding. In addition, there may be past inbreeding that is not detected with pedigree information that can be calculated through genomic inbreeding coefficients [15].

The aim of this study was to identify inbreeding coefficient (F) estimators in a small Holstein population that could be used in genetic improvement programs. Inbreeding coefficients were calculated using different methodologies and sources of information such as runs of homozygosity (Froh) in different lengths, genomic inbreeding coefficients (Fgeno), the genomic relationship matrix (Fmg) and pedigree information (Fped). Correlations among Froh, Fgeno, Fped and Fmg in the Mexican Holstein population were calculated. The behavior of inbreeding coefficients, based on the origin of the evaluated individuals’ parents, was calculated with the objective of evaluating the differences in F when the germplasm came from different countries and whether the different measures were capable of identifying such inbreeding equally among the methods of F. The Froh was also calculated utilizing lengths of ROH from 1 to 2 Mb, 2 to 4 Mb, 4 to 8 Mb, 8 to 16 Mb and >16 Mb to identify the length of ROH that is best associated with Froh with the other coefficients of inbreeding.

## 2. Materials and Methods

To estimate Fped, information based on pedigree was used, which included 212,665 Holstein animals. The number of equivalent complete generations was calculated to estimate the depth of the pedigree using the Pedig software [16]. To estimate Fped, the recursive modified algorithm which takes into account inbreeding different from zero when the parents were unknown and is included in the program INBUPGF90 [17] was used.

For the estimation of Fgeno, Froh and Fmg, 5918 genotypes of Holstein cows from the Mexican population were used. These were distributed in 36 herds and each animal included genomic information of 100,806 SNP markers previously imputed with FindHap V2, VanRaden, USDA, USA [18]. Genotype quality control was applied and genotypes of animals with a call rate < 0.90 were excluded. For SNP, markers with minor allele frequency (MAF) < 0.02, call rate < 0.90 or *p*-value for Hardy–Weinberg < 0.0001 were excluded.

To define ROH, all runs with a minimum length of 1 Mb and at least 10 SNP were included. With these parameters, the risk of including short ROH was avoided, which can be frequent because of linkage disequilibrium (DL) [11]; additionally, one heterozygote SNP per run, five missing genotypes, a minimum density of 1 SNP by 100 kb and a maximum gap between SNP of 500 kb were allowed [19].

To calculate Froh, the methodology used was that proposed by Mcquillan [8], which considers $F_{roh}^{i}=\Sigma L_{roh}^{i}/\;L_{auto}$, where $F_{roh}^{i}$ is the inbreeding coefficient of the individual *i* calculated with ROH; $\Sigma L_{roh}^{i}$ is the total sum of the ROH segments of an individual *i* above a specified minimum length, > 1 Mb in this case; $L_{auto}$ is the length of the autosomal genome covered by SNPs, including centromeres.

The G matrix was calculated using the following formula: $G=\frac{ZZ’}{2\sum P\left(1-P\right)}$*,* where Z, is a matrix containing the subtraction of a base population allele frequency from the given marker values and *p* is the uniform base population allele frequency set at 0.5 due to the fact that the Fmg is better calculated with this allele frequency than estimating it from a base population and [20,21]. The program PREGSF90 from the BLUPF90 v1.70 software (University of Georgia, Athens, GA, USA) was used to calculate G [22]. Fmg was calculated as the diagonal of G minus 1 [20].

To estimate Fgeno, the following formula was used: $f_{geno}=\frac{HO-HE}{NM-HE}$, where HO is the number of observed homozygotic markers; HE is the number of expected homozygotes; NM is the number of markers in each individual [13].

The analyses of ROH and Fgeno were performed with the bioinformatics platform Golden Helix^®^ Variation Suite v.8.8.3., Bozeman, MT, USA [23].

The comparisons between the different estimated inbreeding coefficients were made through the estimation of Pearson’s correlations [24] among coefficients [25], including only the 4277 animals with all four estimations in the analysis (Fgeno, Fped, Froh and Fmg).

In addition, the correlations of Fgeno, Fped, and Fmg with Froh calculated with different ROH lengths were evaluated, with the aim of determining which length of ROH to calculate the Froh was best associated with the inbreeding coefficients. The RHO lengths studied were divided into five classes, from 1 to 2 Mb, 2 to 4 Mb, 4 to 8 Mb, 8 to 16 Mb and >16 Mb, and included 3759 animals out of the total 4277 because animals that did not have the four inbreeding coefficients of the five classes were eliminated.

Additionally, to evaluate the relationship between inbreeding and country of origin of the animal’s parents used in this study and the influence in the population, analyses were performed for countries of origin of sires and dams with more than 20 progenies in Mexico.

It might be expected that the progeny of parents from different countries would have different levels of inbreeding depending on the level of selection in each country of origin, and different expectations of future inbreeding due to the different participation of exporting countries in the genetic makeup of the population, but it is of interest to determine if the different methods for calculating F can better identify the changes in each group of animals.

Finally, the trends of the four inbreeding coefficients by birth year from 2005 to 2017 were evaluated. To prevent problems due to the difference in scales between the birth years and the inbreeding coefficients, birth years were assigned values from 1 to 13. Polynomial regressions [26] were fit to the inbreeding coefficients with the R v3.6.1 [27] software, R Foundation for statistical computing, Vienna, Austria.

## 3. Results and Discussion

### 3.1. Fped and Its Relation with Genomic Inbreeding Coefficients

The number of complete generations calculated with the Pedig Software [16] was seven generations, and the Fped value calculated for this population was 3.11 ± 2.30% (Table 1), which is lower than published values in other populations of the same breed. In Spain, an average of 4.2% was reported [3] and 4.4% in the case of Italy [28].

Fgeno values averaged −0.02 ± 3.55%, values that were low compared to Fped. The correlation between Fped and Fgeno was 0.39, a relationship similar in comparison to the one reported in Holstein bulls in China, where Zhang et al. [29] determined a correlation of 0.38.

The correlation between Fped and Froh was low (0.30) compared with other studies, where correlations were of 0.82 [29] between these coefficients, with a minimum of 20 SNP per RHO have been found. The discrepancies between the results of different studies can be due to differences in the structure of the populations, the parameters used to define ROH, the accuracy and depth of the pedigrees or the effect of ROH’s length, a topic that will be dealt with later in this paper.

In this study, the correlation calculated between Fped and Fmg was of 0.41. In the study conducted by Zhang et al. [29], a negative but not significant correlation (−0.20) between these coefficients was reported in the same cattle breed of our study; additionally, in the same study, the author calculated correlations of −0.18 (*p* < 0.01) for Jersey cattle and 0.36 (*p* < 0.01) for the Danish Red, a Danish native breed; the authors attributed the moderate correlation in the Danish Red to the fact that this breed has a heterozygosity level that is higher than the Jersey or Holstein breeds.

### 3.2. Froh and the Genome Proportion with ROH

The average length of the ROH was 4.8 ± 0.78 Mb with a minimum length of 1.54 Mb and a maximum of 100.56 Mb, and an average number of 47 ± 10.73 ROH per animal. The average length of the genome covered by ROH was 276.89 Mb for the population in this study, similar to that of Holstein cattle in the USA, where two studies demonstrated a covered genome’s length of 290.6 Mb and 299.6 Mb [30,31], which could be the reason for a higher estimation of the inbreeding degree for animals descending from dams of USA origin. This may be because the registered Holstein population of Mexico presents a high genetic relationship with the population in the USA [32].

The Froh calculated in this study was (2.72 ± 0.71%) which is lower than the one calculated for Holstein cattle from Italy and the USA, 3.8 ± 2.1% and 4.2 ± 2.3%, respectively [5]. On the other hand, higher Froh values have been published by Rodríguez-Ramilo et al. [3] in Holstein animals from Spain (7.7%). The differences in Froh estimates can be attributed mainly to the effective size of the population, missing pedigree information or the definitions of ROH used [5,21]. Froh is a good estimator of the homozygosity in the genome of each individual but has its disadvantages when calculating the homozygosity in the population based on low-density arrays due to the relationship between density and the length of the ROH determined [33].

### 3.3. Froh with Different Lengths of ROH and Its Relationship with Other Inbreeding Coefficients

The largest proportion of ROH found were those from 1 to 2 Mb with 51.32%, followed by those of 2 to 4 Mb with 22%, with the least being those greater than 16 Mb with 15% (Table 2). The highest number of short ROH is related to ancient inbreeding [28].

The correlation calculated between Froh and Fgeno was 0.82, higher than the results reported by Zhang et al. [29] who found a correlation of 0.61 between the same coefficients. Mastrangelo et al. [5] reported higher correlation values between Froh and Fgeno (0.89), similar to the ones found in this study, and the authors attributed this to recent inbreeding in the Italian Holstein population.

Table 3 shows the correlation coefficients between Fgeno and Fped with Froh with different lengths of ROH. The correlations increased for all coefficients as ROH lengths increased, particularly when the runs were higher than 16 Mb.

The correlation between Froh calculated with different lengths of ROH with the other coefficients increased when the length of the runs was greater than 2 Mb for Fgeno and Fmg, and with Fped increased when the length of the runs was greater than 8 Mb (Table 3). Makanjuola et al. [34] reported similar results in a study of Canadian Holstein cattle, with increasing correlations between Froh and Fmg from ≈0.20 with Froh _2–4_ to ≈0.70 with Froh _>16_. The trends in the correlation between Fped with Froh calculated with the different lengths of ROH was similar to that reported by Makanjuola et al. [34] who found correlations with a negative level (≈−0.10) with Froh_1–2_ and positive correlations (≈0.40) with Froh _>16_. However, they did not find the same correlation trend between Froh (calculated with the five ROH lengths) with Fped (calculated with seven generations of common ancestors) probably because the pedigree only includes ancient inbreeding.

In contrast, a simulation study in a population of 1000 individuals, Caballero et al. [35] reported a correlation of 0.66 between Froh and Fgeno when the length of the ROH was greater than 5 Mb, and of 0.82 when the length of the ROH was greater than 1 Mb. The authors suggested that this could be explained because short ROH may not fully reflect the proportion of the genome which is identical by descent, overestimating the true genomic inbreeding.

**Figure 1:**
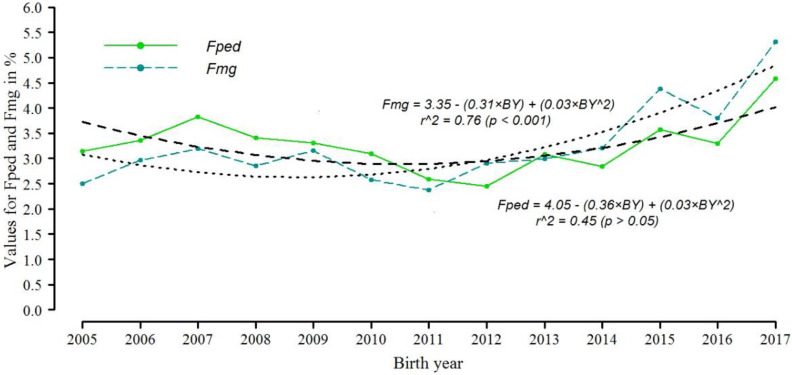
Distribution of inbreeding coefficients calculated through pedigree (Fped) and calculated through genomic relationship matrix (Fmg), from 2005 to 2017, with trends lines. BY: Birth year recoded from 1 to 13, BY^2: Squared recoded birth year.

**Figure 2:**
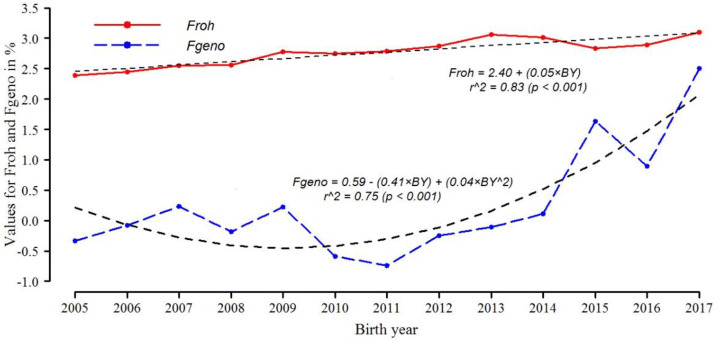
Distribution of inbreeding coefficients calculated by Runs of Homozygosity (Froh) and calculated by the number of homozygotic SNP markers observed and expected (Fgeno), from 2005 to 2017, with trend lines. BY: Birth year recoded from 1 to 13, BY^2: Squared recoded birth year.

Ferenčaković et al. [13] reported a similar trend as the one found in this study. They calculated a correlation between Froh and Fped of 0.50 with ROH > 1 Mb and 0.66 with ROH > 16 Mb in Brown Swiss cattle. The increased correlation in our study can probably be attributed to the fact that the population shows recent inbreeding (Figure 1 and Figure 2), confirmed by the frequency of large RHO [29]. Gurgul et al. [36] obtained a low correlation between Fped and Froh (0.295) with an ROH length of 4 Mb with five pedigree generations and found even lower correlations (0.243) when ROH were longer than 16 Mb, explaining that the relationship between Fped and Froh could be affected by the number of generations included in the pedigree since Froh with long ROH reflects the recent inbreeding of animals.

### 3.4. Fmg and Its Relationship with the Other Inbreeding Coefficients

The correlation found between Fmg and Fgeno (0.96) was higher than the one obtained between Fmg and Fped (0.41), because Fmg and Fgeno use individual genetic markers for their estimation while Fped does not utilize genomic information. The correlation reported between Fmg and Froh has been very variable. While the correlation found in this study was high (0.82), and was different from Mastrangelo et al. [5], who calculated Fmg values of 0.042 in Italy’s Holstein cattle and a non-significant correlation with Froh of 0.18. Bjelland et al. [21] estimated a correlation between Fmg and Froh similar to ours which was 0.81 in the USA’s Holstein cattle. Caution should be exercised when using Fmg because Villanueva et al. [37] mentioned that Fmg can lead to inconsistent results in terms of the gain and loss of genetic variability, and, thus, to misleading interpretations, and do not always provide a useful measure of inbreeding.

### 3.5. Fgeno and the Possibility of Mating with Different Populations

In this study, Fgeno had a negative estimate similar to that found by Mastrangelo et al. [5] who reported values for Fgeno of −1.4% in Italy’s Holstein breed. Of the analyzed animals, 2361 (55.20%) had a negative value for Fgeno, with an average of −2.34%, a value that could indicate that the estimated population comes from distant families or because the animals are more heterozygous than the population average [13].

### 3.6. Influence of the Origin Parents’ Country on The Level of Inbreeding in the Offspring

Table 4 analyzes the influence of the parent’s country of origin, showing the inbreeding coefficient values for animals with positive Fgeno, Fped, Froh and Fmg (1852 animals), comparing animals with parents from Canada (CAN), the United States of America (USA), the Netherlands (NLD) and Mexico (MEX).

Animals that descended from parents from NLD showed lower values of Fgeno (2.22 ± 1.92%) than the rest, probably due to the reduced number of animals descending from these parents in the pedigree and the fact that they come from a more genetically distant population in the study. On the other hand, animals with Mexican parents had the highest Fped (4.87 ± 3.46%), which could be attributed to the intensive use of local animals inside the population or in particular subpopulations.

Van Doormaal, in 2016, cited by Andere et al. [38], reported an Fped value in a Holstein cattle population from Canada of 7.10%, which is higher than the one found in this study (3.48%) for animals descending from Canadian parents; which, altogether with the results obtained for Froh (3.40%) and Fgeno (2.95%), suggests a higher inbreeding average for Canadian parent’s offspring compared to the other countries in this study with the exception of Mexico.

The descendants from CAN or the USA presented higher Froh values than animals with ancestors from Mexico. This could be because in these countries the intensity of selection is greater than in Mexico. García-Ruiz et al. [39] reported an increase in the inbreeding calculated by pedigree from 4.3% in the year 2000 to 5.8% in the year 2010 in Holstein cattle from the USA, and from 5.8% to 6.4% in sires, which reflects an increase in inbreeding in the last few years for this population.

### 3.7. Inbreeding Coefficient Trends

Figure 1 shows Fped and Fmg’s trends for the years from 2005 to 2017. Although the trends are similar in both, the annual change of Fped is not statistically significant (*p* > 0.05) with a coefficient of determination (r^2^) of 0.45, different from the coefficient of determination (r^2^) for Fmg which was 0.76. The differences in trends and the coefficient of determination are related to the low correlation between Fped and Fmg which was 0.41.

Figure 2 illustrates the annual accumulation of inbreeding measured by Froh and Fgeno. Although the correlation between Froh and Fgeno was high (0.82), the trend lines are dissimilar, with r^2^ = 0.83 for Froh and 2.08 r^2^ = 0.75 for Fgeno. The high correlation between Fgeno and Fmg (0.96) and similar slopes to B_1_ (−0.41 and −0.31 respectively) were expected because both use frequencies of homozygous SNP markers and heterozygotes.

Considering that the correlation between Froh based on large ROH and other F measures was higher than the other lengths (Table 3), and that this Froh is associated with recent inbreeding, it follows that the recent −inbreeding increase in Fgeno and Fmg should be expected.

## 4. Conclusions

In small populations, inbreeding based on genomic information is consistent among the different estimates, however, their correlation with Fped is low. Nevertheless, Froh and Fgeno supply additional information such as how far back inbreeding was present in the population. In this study, Froh showed recent inbreeding in the Mexican Holstein population and Fgeno showed the possibility that the ancestors of the evaluated animals had been mated with animals of different breeds or with animals genetically distant. Froh presented higher correlations with the other inbreeding coefficients as the length of the ROH increased. Finally, differences were observed between the ancestor’s countries of origin regardless of the method of calculating the inbreeding coefficient.

## Figures and Tables

**Table 1:** Average inbreeding coefficient (F% ± SD) and correlations (±SE) among the inbreeding calculation methods (below diagonal; *p* < 0.0001).

F%	Fped 3.11 ± 2.30	Fgeno −0.02 ± 3.55	Froh 2.77 ± 0.71	Fmg 3.03 ± 3.05
Fped	1			
Fgeno	0.39 ± 0.013	1		
Froh	0.30 ± 0.014	0.82 ± 0.005	1	
Fmg	0.41 ± 0.013	0.96 ± 0.001	0.82 ± 0.005	1

F%: inbreeding coefficient in percentage, Fped: Inbreeding coefficient calculated through pedigree, Fgeno: Inbreeding coefficient calculated by the number of homozygotic SNP markers observed and expected, Fmg: Inbreeding coefficient calculated through genomic relationship matrix. Froh: Inbreeding coefficient calculated by runs of homozygosity, Number of observations with all coefficients: 4277, SD: Standard deviation, SE: Standard error.

**Table 2:** Number and percentage of ROH by length.

Length Mb	No. ROH	%
1–2	215,175	51.32
2–4	93,596	22.32
4–8	61,329	14.63
8–16	34,375	8.20
>16	14,812	3.53

Mb: Megabase, ROH: Runs of Homozygosity.

**Table 3:** Correlations (±SE) among the inbreeding coefficients between Fgeno, Fmg and Fped with Froh with different lengths of ROH.

Froh with Different Lengths of ROH					
	1–2 Mb (Froh _1–2_)	2–4 Mb (Froh _2–4_)	4–8 Mb (Froh _4–8_)	8–16 Mb (Froh _8–16_)	>16 Mb (Froh _>16_)
N° of animals	3759				
Fgeno	−0.01 ± 0.016	0.14 ± 0.016 *	0.30 ± 0.015 *	0.46 ± 0.013 *	0.71 ± 0.008 *
Fped	−0.03 ± 0.016	−0.02 ± 0.016	0.05 ± 0.016	0.15 ± 0.016 *	0.34 ± 0.014 *
Fmg	0.01 ± 0.016	0.16 ± 0.016 *	0.30 ± 0.015 *	0.46 ± 0.013 *	0.69 ± 0.008 *

Mb: Megabase, ROH: Runs of homozygosity, Fmg: Inbreeding coefficient calculated through genomic relationship matrix, Fgeno: Inbreeding coefficient calculated from the number of homozygotic SNP markers observed and expected, Froh: Inbreeding coefficient calculated from the number of Homozygotic SNP markers, Fped: Inbreeding coefficient calculated through pedigree, SE: Standard error, * Statistically significant correlations (*p* < 0.0001).

**Table 4:** Inbreeding coefficients in % (±SE) for animals with positive Fgeno, grouped by sire and dam’s country of origin.

Sire’s Country of Origin	No of Calves	Fgeno	Fped	Froh	Fmg
MEX	405	3.35 ± 0.19	4.87 ± 0.16	3.07 ± 0.04	5.60 ± 0.19
USA	1095	2.69 ± 0.08	3.33 ± 0.08	3.31 ± 0.02	5.31 ± 0.07
CAN	282	2.95 ± 0.16	3.48 ± 0.15	3.40 ± 0.04	5.55 ± 0.15
NLD	67	2.22 ± 0.23	3.55 ± 0.23	3.18 ± 0.06	4.99 ± 0.21
Dam’s country of origin					
MEX	1599	2.85 ± 0.07	3.68 ± 0.07	3.26 ± 0.02	5.38 ± 0.07
USA	32	2.33 ± 0.34	4.36 ± 0.38	3.17 ± 0.10	5.13 ± 0.29
CAN	23	3.11 ± 0.53	3.39 ± 0.48	3.19 ± 0.14	5.85 ± 0.47

Only values for groups with more than 20 calves are shown. MEX: Mexico, USA: United States of America, CAN: Canada, NLD: Netherlands. Fped: Inbreeding coefficient calculated through pedigree, Fgeno: Inbreeding coefficient calculated by the number of homozygotic SNP markers observed and expected, Froh: Inbreeding coefficient calculated from runs of homozygotic, Fmg: Inbreeding coefficient calculated through the genomic relationship matrix.
